# Characterisation of the in-vivo miRNA landscape in Drosophila ribonuclease mutants reveals Pacman-mediated regulation of the highly conserved let-7 cluster during apoptotic processes

**DOI:** 10.3389/fgene.2024.1272689

**Published:** 2024-02-20

**Authors:** Elisa I. M. Bernard, Benjamin P. Towler, Oliver M. Rogoyski, Sarah F. Newbury

**Affiliations:** ^1^ Brighton and Sussex Medical School, University of Sussex, Brighton, United Kingdom; ^2^ School of Life Sciences, University of Sussex, Brighton, United Kingdom

**Keywords:** RNA turnover, XRN1, DIS3L2, apoptosis, miRNA stability

## Abstract

The control of gene expression is a fundamental process essential for correct development and to maintain homeostasis. Many post-transcriptional mechanisms exist to maintain the correct levels of each RNA transcript within the cell. Controlled and targeted cytoplasmic RNA degradation is one such mechanism with the 5′-3′ exoribonuclease Pacman (XRN1) and the 3′-5′ exoribonuclease Dis3L2 playing crucial roles. Loss of function mutations in either Pacman or Dis3L2 have been demonstrated to result in distinct phenotypes, and both have been implicated in human disease. One mechanism by which gene expression is controlled is through the function of miRNAs which have been shown to be crucial for the control of almost all cellular processes. Although the biogenesis and mechanisms of action of miRNAs have been comprehensively studied, the mechanisms regulating their own turnover are not well understood. Here we characterise the miRNA landscape in a natural developing tissue, the *Drosophila melanogaster* wing imaginal disc, and assess the importance of Pacman and Dis3L2 on the abundance of miRNAs. We reveal a complex landscape of miRNA expression and show that whilst a null mutation in *dis3L2* has a minimal effect on the miRNA expression profile, loss of Pacman has a profound effect with a third of all detected miRNAs demonstrating Pacman sensitivity. We also reveal a role for Pacman in regulating the highly conserved *let-7* cluster (containing *miR-100, let-7* and *miR-125*) and present a genetic model outlining a positive feedback loop regulated by Pacman which enhances our understanding of the apoptotic phenotype observed in Pacman mutants.

## Introduction

MicroRNAs (miRNAs) are a class of small non-coding RNAs that serve as regulators of gene expression in eukaryotic cells. miRNAs are 20–22 nucleotide (nt) long transcripts which have been shown to regulate the expression of a diverse range of RNAs and play a role in virtually every aspect of development. Since each miRNA can target many mRNAs, and mRNAs can include binding sites for a number of miRNAs, the impact of miRNA activity on the transcriptome and cellular function is profound and complex (Brennecke et al., 2003; Ge et al., 2012; Zhang and Cohen, 2013; Aparicio et al., 2015; Bolin et al., 2016; Kane et al., 2018; Volin et al., 2018; Yu et al., 2020; Bejarano et al., 2021). In addition, dysregulation of a single miRNA can result in dramatic phenotypes in model organisms such as *Drosophila* and mice (Schertel et al., 2012; Towler et al., 2015; Bartel, 2018). For example, overexpression of *miR-252–5p* in the *Drosophila* wing pouch results in severe wing defects (Towler et al., 2015). Altered miRNA expression is also known to contribute to the pathogenesis of human diseases including cancer (Mendell and Olson, 2012).

miRNAs are usually transcribed as long primary transcripts (pri-miRNAs) which undergo a series of processing events to produce mature miRNAs. The genomic location of miRNAs shows great variety; they can be localised within genes encoding proteins, snoRNAs, lncRNAs, tRNAs or in separate transcription units and are often present in clusters containing multiple miRNAs of the same family (Soleimani et al., 2020). In the canonical pathway, pri-miRNAs form hairpin structures which are cleaved co-transcriptionally by the microprocessor complex comprising DGCR8 (Pasha in invertebrates) and Drosha to release the pre-miRNA hairpin loop which is then exported to the cytoplasm. Dicer (Dicer-1 in *Drosophila*) cleaves the hairpin to produce the miRNA duplex, which comprises two imperfectly complementary strands of around 22 nt. Finally, one strand of the miRNA duplex is loaded onto an Argonaute protein (Ago; Ago1 in *Drosophila*) which is part of the miRNA-induced silencing complex (RISC). Alternatively, miRNAs (termed mirtrons) originate from introns of protein-coding genes (Ruby et al., 2007) but differ in their biogenesis in that splicing, rather than Drosha, defines the pre-miRNA. After splicing of the mirtron precursor, the intron lariat is de-branched to allow folding and loading onto Dicer. Some mirtrons, termed “tailed mirtrons,” are derived from longer pri-miRNAs which need to be trimmed before Dicer binding and cleavage (Flynt et al., 2010). The processed mirtron is then loaded onto an Ago protein to provide an active complex, which then interacts with the target RNA.

miRNAs have been reported to be extremely stable and exhibit slower turnover than most RNAs. This is thought to be because the 5′ and 3′ ends of each miRNA are buried within the AGO protein, shielding them from ribonucleases (Sheu-Gruttadauria et al., 2019a; Sheu-Gruttadauria et al., 2019b). However, miRNAs with short half-lives have been observed and rates of turnover can also vary in different tissues and during transitions. For example, metabolic labelling of *Drosophila* S2 cells has shown that half-lives can vary from <2 h (e.g., *miR-12-5p*) to more than 24 h (e.g., *bantam-3p*) (Reichholf et al., 2019). These data suggest that there are specific mechanisms which promote miRNA degradation in response to cellular or developmental cues.

Despite their importance in post-transcriptional regulation, little is known about the exoribonucleases responsible for degrading specific miRNAs. In our previous work using *Drosophila* wing imaginal discs, we showed that specific miRNAs (e.g., *miR-277-3p*) are sensitive to depletion of the 5′-3′ exoribonuclease XRN1 (Pacman) (Jones et al., 2013). Cytoplasmic XRN-1 was also shown to be involved in mature miRNA turnover in *C. elegans* irrespective of whether the miRNA was associated with Ago (Chatterjee et al., 2011). It is possible that XRN-1 is recruited to the miRNAs by the decapping scavenger enzyme DCS-1 (Bosse et al., 2013). In another study from our lab, the exosome-associated 3′-5′ exoribonuclease Dis3 was shown to regulate the levels of *miR-277-3p* and *miR-252-5p* in wing imaginal discs (Towler et al., 2015). Mammalian cell lines have been used to demonstrate a role for DIS3L2 in miRNA and pre-miRNA decay following their terminal tailing including uridylation by uridylyltransferases such as TUT4 and TUT7 (Heo et al., 2009; Heo et al., 2012; Chang et al., 2013; Ustianenko et al., 2016; Shukla et al., 2019). However, this tailing does not necessarily lead to miRNA degradation (Yang et al., 2020). An example of tail-mediated miRNA decay includes maternally deposited miRNAs in *Drosophila*, which can be cleared during the maternal to zygotic transition in a process that involves adenylation by the non-canonical polyA polymerase Wispy (Lee et al., 2014). These data suggest that specific miRNAs are sensitive to depletion of specific ribonucleases.

Our previous work using *Drosophila* wing imaginal discs has shown that disruption of the major cytoplasmic decay complexes results in contrasting phenotypes in natural tissues (Jones et al., 2013; Waldron et al., 2015; Jones et al., 2016; Towler et al., 2016; Towler et al., 2020). Depletion of the 5′-3′ exoribonuclease Pacman (XRN1) results in increased developmental delay and apoptosis (Waldron et al., 2015; Jones et al., 2016), whereas depletion of Dis3L2 results in enhanced proliferation (Towler et al., 2020). Using RNA-sequencing we have identified some of the ribonuclease sensitive mRNA transcripts that underpin the mutant phenotypes observed. *Dilp8* is upregulated in *pacman* mutants and drives developmental delay (Waldron et al., 2015; Jones et al., 2016), whereas *idgf2* contributes to tissue overgrowth in *dis3L2* mutants (Towler et al., 2020). However, the miRNAs specifically sensitive to Pacman and/or Dis3L2 within these natural tissues have not been identified, and their contribution to the observed loss of function phenotypes is as yet unknown.

In this investigation, we characterised the *in vivo* miRNA landscape using *Drosophila* wing imaginal discs and assessed their susceptibility to Pacman or Dis3L2 null mutations. We show that lncRNAs hosting miRNAs are both more abundant and longer than the genome average. We demonstrate that a greater proportion of mature miRNAs, including a group of mirtrons, are sensitive to Pacman compared to Dis3L2 suggesting that the 5′-3′ degradation pathway is important in maintaining the correct levels of these miRNAs. Importantly, we demonstrate a potential role for Pacman in regulating the highly conserved and developmentally important *let-7* miRNA cluster. A model to explain the contribution of Pacman and its effect on the *let-7* cluster in apoptosis, which incorporates our previous data, is presented.

## Materials and methods

### *Drosophila* husbandry

*Drosophila* stocks were cultured on standard media and all experiments were performed at 25°C. The *dis3L2* ^*12*^ null mutant and its isogenic control were produced in (Towler et al., 2020). The *pcm* ^*14*^ null mutant and its isogenic control were generated in (Waldron et al., 2015). The following stocks were obtained from Bloomington Stock Center: *nubbin-GAL4* (stock 25754; P{UAS-Dcr-2.D}1, w1^118^; P{GawB}nub-AC-62), 69B-GAL4 , w*;; P{GawB}69B, Df(3L)H99 kni^ri-1^ p^p^/TM3 Sb^1^, UAS-rpr, w^1118^; P{w[+mC]=UAS-rpr.C}14, dilp8-minos, UAS-dilp8 was a kind gift from Dr Pierre Leopold.

### Wing imaginal disc dissection

Imaginal discs were dissected from 3^rd^ instar wandering larvae; for *pcm* ^*WT*^, *dis3L2* ^*12*^ and *dis3L2* ^*WT*^ 90 wing discs were dissected and pooled for each of the 4 replicates; whereas 180 discs were collected for each pool for the *pcm* ^*14*^ mutant, as they are 45% the size of controls (Waldron et al., 2015). Since *pcm* is on the X chromosome and the *pcm* ^*14*^ mutation is completely lethal at the pupal stage, the only mutant larvae available are hemizygous males. To account for any sex-specific differences in gene expression, male *pcm* ^*WT*^ larvae were collected. To allow accurate developmental staging and avoid overcrowding, 3 h egg lays were performed. 120 h after egg lay, wandering 3^rd^ instar (L3) larvae were collected and dissected in Ringers solution (3 mM CaCl_2_, 182 mM KCl, 46 mM NaCl, 10 mM Tris pH7.2) under a Nikon SMZ800 dissection microscope and flash frozen in liquid nitrogen. *Dilp8-minos* expression was assessed in 120 h wing imaginal discs with tissues dissected and mounted as in (Towler et al., 2020) and imaged on a Leica SP8 confocal microscope.

### miRNA-seq and bioinformatic analysis

RNA extraction was performed on between 60 and 180 120 h wing imaginal discs using the miRNeasy Micro Kit (Qiagen #217084) according to manufacturer’s instructions. *2S rRNA* depletion was performed essentially as described in (Aspden et al., 2014). Briefly, 750 pmoles of *2S rRNA* oligo was bound to 60 µL of MyOne Streptavidin C1 Dynabeads (Thermo Fisher #65001) for each sample in Tris-EDTA buffer. 30 μL of oligo-bound beads were used to deplete 400 ng of sample RNA. Two rounds of depletion were performed. The RNA was then precipitated and its integrity verified using an RNA 6000 nano chip (Agilent, #5067–1511) on a Bioanalyser 2100. sRNA-seq libraries were made using the QIAseq^®^ miRNA Library Preparation Kit (Qiagen #331502), according to the manufacturer’s instructions, using 6 µL of 2S-depleted RNA. Library quality was assessed using a DNA High Sensitivity chip (Agilent #5067–4626) on a Bioanalyser 2100. Libraries were sequenced on a NextSeq500 at Leeds Genomics obtaining a total of 326,514,877 reads with individual sample coverage ranging from 15.3 to 23.9 million reads.

To analyse the sRNA-seq data, miRDeep2 was used to build a genome with the dmel-all-chromosomes-6.29.fasta file from FlyBase (Consortium et al., 2018). Reads were mapped in miRDeep2 (Friedlander et al., 2012), with the parameters -h, -k, -l17 and -m and subsequently quantified using miRDeep2. Differential expression analysis was performed using DESeq2 using the default parameters in R. The data was independently analysed with sRNAbench from the sRNAtoolbox suite mapping (Aparicio-Puerta et al., 2019; Aparicio-Puerta et al., 2022) to BDGR6 (Hoskins et al., 2015) taking into account UMIs incorporated during the library preparation. To be classified as differentially expressed, miRNAs had to display a fold change >1.5 and padj<0.05 in the miRDeep2 analysis together with 4 of the 5 statistical outputs from sRNAbench. All normalised read count and fold change data presented in this work is from the miRDeep2 pipeline.

### RNA extraction and qRT-PCR

RNA extraction was performed on between 30 and 120, 120 h wing imaginal discs or a single 120 h whole L3 larvae using the miRNeasy Micro Kit (Qiagen #217084) according to manufacturer’s instructions. Reverse transcription for miRNAs and *snoR442* were performed using the TaqMan MiRNA Reverse Transcription Kit (Applied Biosystems #4366596) according to manufacturer’s instructions. All qRT-PCR performed on miRNAs, excluding the RISC-bound experiments, were normalised to *snoR442* which showed consistent expression across all genotypes. Reverse transcription for other transcripts was performed using the High-Capacity cDNA Reverse Transcription Kit (Applied Biosystems #4368814) according to manufacturer’s instructions. qRT-PCR were performed in technical triplicate using the TaqMan Fast Universal PCR Master Mix (Thermo Fisher #4352042) according to manufacturer’s instructions in either a ViiA7 or QuantStudio7 Flex Real-Time PCR machine. All non-miRNA qRT-PCR experiments were normalised to *rpl32* which showed consistent expression across all genotypes. All primers used in this study can be found in Supplemental File S2.

### Isolation of RISC-associated miRNAs

To isolate the miRNAs bound by RISC, the TraPR Small RNA Isolation Kit (Lexogen #128) was used following manufacturer’s instructions. 60 *pcm* ^*WT*^ and 120 *pcm* ^*14*^ wing imaginal discs were used as starting material. The levels of the miRNAs were measured using TaqMan miRNA assays and normalised to the levels of *miR-275-5p* which showed consistent expression across all genotypes.

### Statistical analysis

All statistical analyses were performed in either R v4.2.2 or GraphPad Prism 8. Two-sided two-sample t-tests were used to compare the means of single test groups to single control groups. If multiple comparisons were required, a one-way ANOVA was performed with a post-test to compare the means of each possible pair of samples. Outputs from statistical analyses can be found in Supplemental File S1 and Supplementary Tables S1–S5.

## Results

### Global expression analysis characterises the microRNA landscape in *Drosophila* wing imaginal discs

**FIGURE 1:**
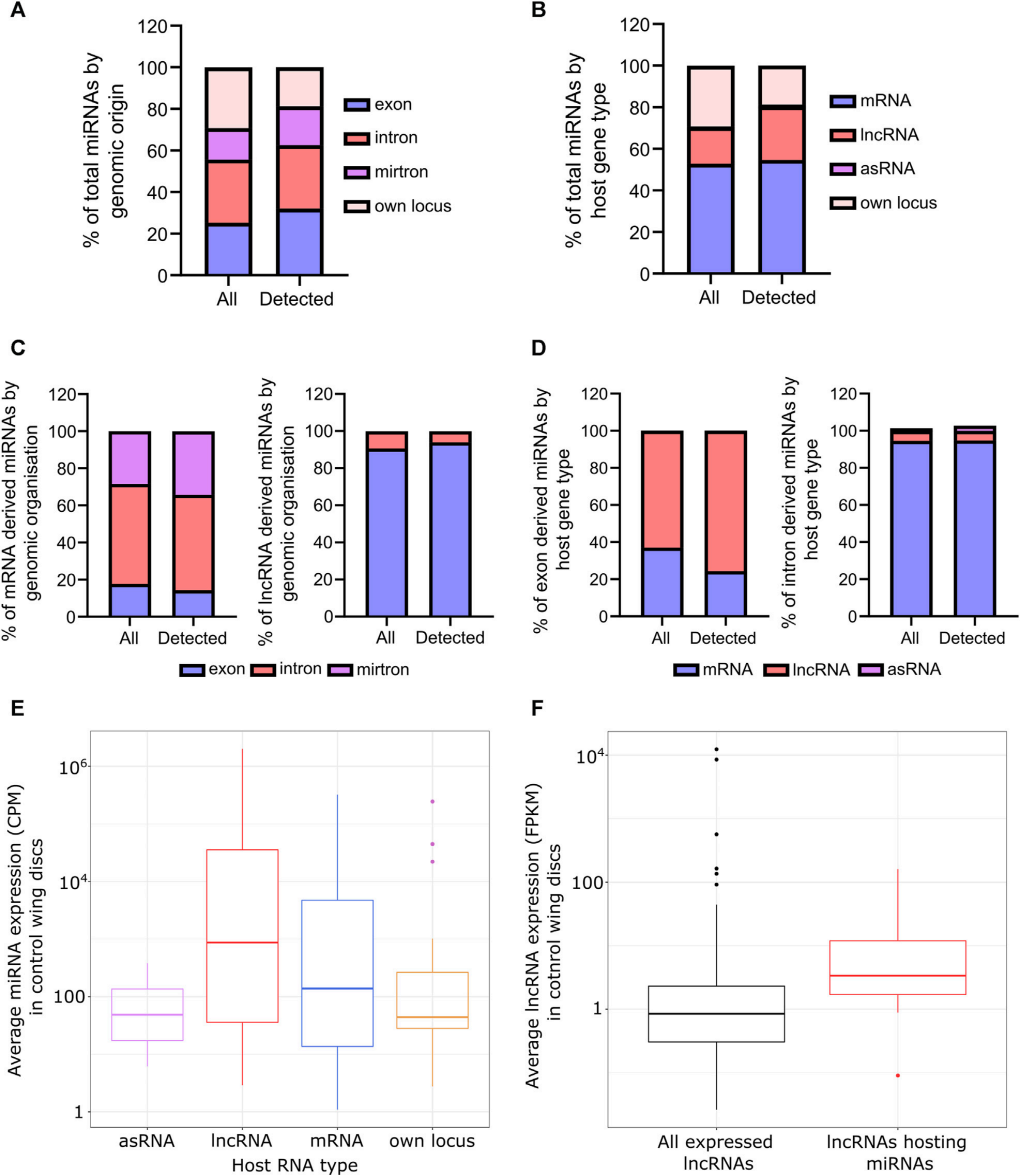
Overview of the miRNAs expressed in wing imaginal discs. **(A)** Genomic origin of all annotated miRNAs (all) and miRNAs reliably detected in all control replicates (detected). **(B)** Proportion of all annotated miRNAs (all) compared to those reliably detected miRNAs by the nature of their host RNA. **(C)** miRNAs derived from mRNAs are largely intronic whilst those from lncRNAs predominantly reside in exons. Those from mRNAs tend to reside in the UTR of their host. **(D)** As **C**, but from the host RNA perspective. **(E)** miRNAs derived from host RNAs tend to be more abundant than those transcribed from their own locus. **(F)** lncRNAs encoding miRNAs are more abundant than the average expression of lncRNAs in the wing disc (*p* = 0.001253). 508 lncRNAs in total detected in wing imaginal discs from Towler et al. 2020.

To provide a comprehensive assessment of the role of Pacman and Dis3L2 in regulating miRNAs, which also may contribute to the observed phenotypes, we performed small RNA sequencing on Pacman null (*pcm* ^*14*^) and Dis3L2 null (*dis3L2* ^*12*^) mutant wing imaginal discs from wandering L3 larvae along with their isogenic control lines (*pcm* ^*WT*^ and *dis3L2* ^WT^) (Waldron et al., 2015; Towler et al., 2020). We first used this data as a starting point to reveal, for the first time, the overall miRNA expression landscape, in terms of numbers, expression levels and genomic origin of miRNAs in wild-type *Drosophila* wing imaginal discs. We identified 201 miRNAs out of 481 annotated *Drosophila* miRNAs (miRbase release 6 v22, (Kozomara et al., 2018)) as confidently and consistently expressed across all 8 control replicates. Expression of miRNAs in these controls were highly correlated (*R* ^2^ = 0.997; *p* < 0.0001; Supplementary Figures S1A, B) demonstrating the technical reliability and consistency of miRNA expression within these tissues. First, we assessed the genomic origin of the detected miRNAs and observed 32% of these originated from exons within host transcripts (Figure 1A). Only 19% were expressed from their own locus, a reduction on the 29% of all annotated miRNAs within the genome transcribed from their own locus, suggesting these miRNAs may be particularly tissue specific. Large intron derived miRNAs accounted for 30% of detected miRNAs whilst 19% of miRNAs were mirtrons, defined as the pre-miRNA taking up the entire intron (Figure 1A). Consistent with a role of lncRNAs as miRNA host transcripts, 26% of miRNAs are processed from lncRNAs and 94% of these are encoded within exons (Figures 1B–D). Strikingly, 55% of miRNAs were hosted within mRNAs, with the majority of these within intronic regions (86%) and miRNAs derived from mRNA exons located in UTRs. This outlines the extensive regulation required to maintain miRNA homeostasis.

Next, we asked if miRNAs from different host transcripts or genomic origins demonstrated expression patterns. We saw that, on average, those derived from host transcripts were more abundant than those from their own locus (Figure 1E; Supplementary Figure S1C) and interestingly, lncRNAs hosting miRNAs were both more abundant and longer than the genome average as calculated from our previous data in the same tissues (Towler et al., 2020) (Figure 1F; Supplementary Figure S1D). The reduced expression in those expressed from their own locus may also contribute to the reduced proportion observed in the wing disc compared to genome annotation.

### Loss of Dis3L2 has a minimal effect on the miRNA landscape

Having characterised the miRNA transcriptome in control tissues we next aimed to assess the effect of different ribonucleases on the wing imaginal disc miRNA landscape with the hypothesis that specific miRNAs may be sensitive to the loss of specific ribonucleases. We hypothesised that if the degradation of a specific miRNA was dependent upon a specific decay pathway, then an increase in expression would be observed in loss of function mutants. Alternatively, a decrease in expression may suggest a role for the ribonuclease in miRNA biogenesis or indirect regulation.

To ask if specific miRNAs are sensitive to loss of Dis3L2 in a natural tissue, we compared the miRNA expression profile in *dis3L2* ^*WT*^ to that in *dis3L2* ^*12*^ null mutant wing imaginal discs using two analysis pipelines, miRDeep2 (Friedlander et al., 2012) and sRNAbench (Aparicio-Puerta et al., 2019; Aparicio-Puerta et al., 2022) with a miRNA requiring a significant change in expression by both pipelines to be considered ribonuclease-sensitive (>1.5 fold change, padj<0.05). A total of 221 miRNAs were detected in all *dis3L2* ^*WT*^ and *dis3L2* ^*12*^ replicates. All processed data can be found in Supplemental File S1.

**FIGURE 2:**
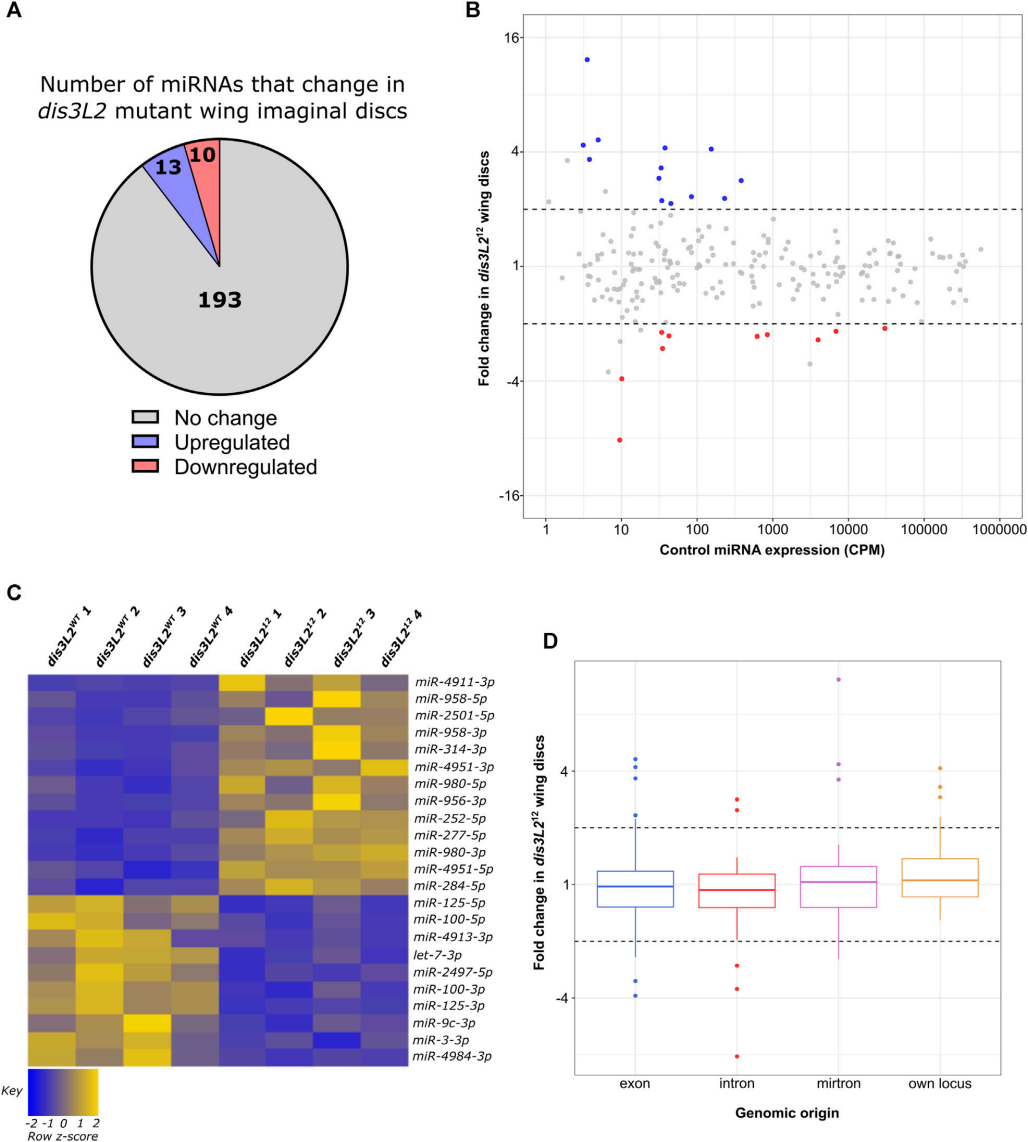
A specific subset of miRNAs are affected by the loss of Dis3L2. **(A)** Proportion of miRNAs significantly dysregulated in *dis3L2* ^*12*^ wing imaginal discs. miRNAs determined as differentially expressed if the fold change is >1.5 and called significant by both analysis pipelines. **(B)** MA plot displaying all miRNAs expressed in wing imaginal discs with upregulated miRNAs in blue and downregulated in red. Dotted lines represent ^±^2-fold change. Displayed CPM and fold change calculated using DESeq2. **(C)** Heatmap representing the expression of differentially expressed miRNAs in all replicates of *dis3L2* ^*WT*^ and *dis3L2* ^*12*^ wing imaginal discs. **(D)** Boxplot displaying fold change of all miRNAs in *dis3L2* ^*12*^ wing discs by genomic origin. Dotted lines represent ^±^2-fold change.

Loss of *dis3L2* resulted in minimal changes in the miRNA expression profile with only 13 (6%) showing upregulation and 10 (5%) showing downregulation in *dis3L2* ^*12*^ tissues (Figures 2A–C). Whilst we observed an increase in expression of miRNAs hosted within asRNAs, this is likely biased by the small number of these miRNAs expressed (2 in total). Therefore, we did not observe any strong trend in miRNA sensitivity to Dis3L2 based on their origin or host RNA (Supplementary Figures S2A, C, D). As we saw an absence of miRNAs transcribed from their own locus in the downregulated pool, we used our previous data set to assess correlation between the sensitivity of the miRNA or its host RNA to Dis3L2; all but 1 miRNA changed in levels independently from their host suggesting these changes in miRNA expression represent independent post-transcriptional regulation rather than a result of regulation of their host transcript (Supplementary Figure S2E).

### Pacman function is required to maintain the miRNA expression profile

**FIGURE 3:**
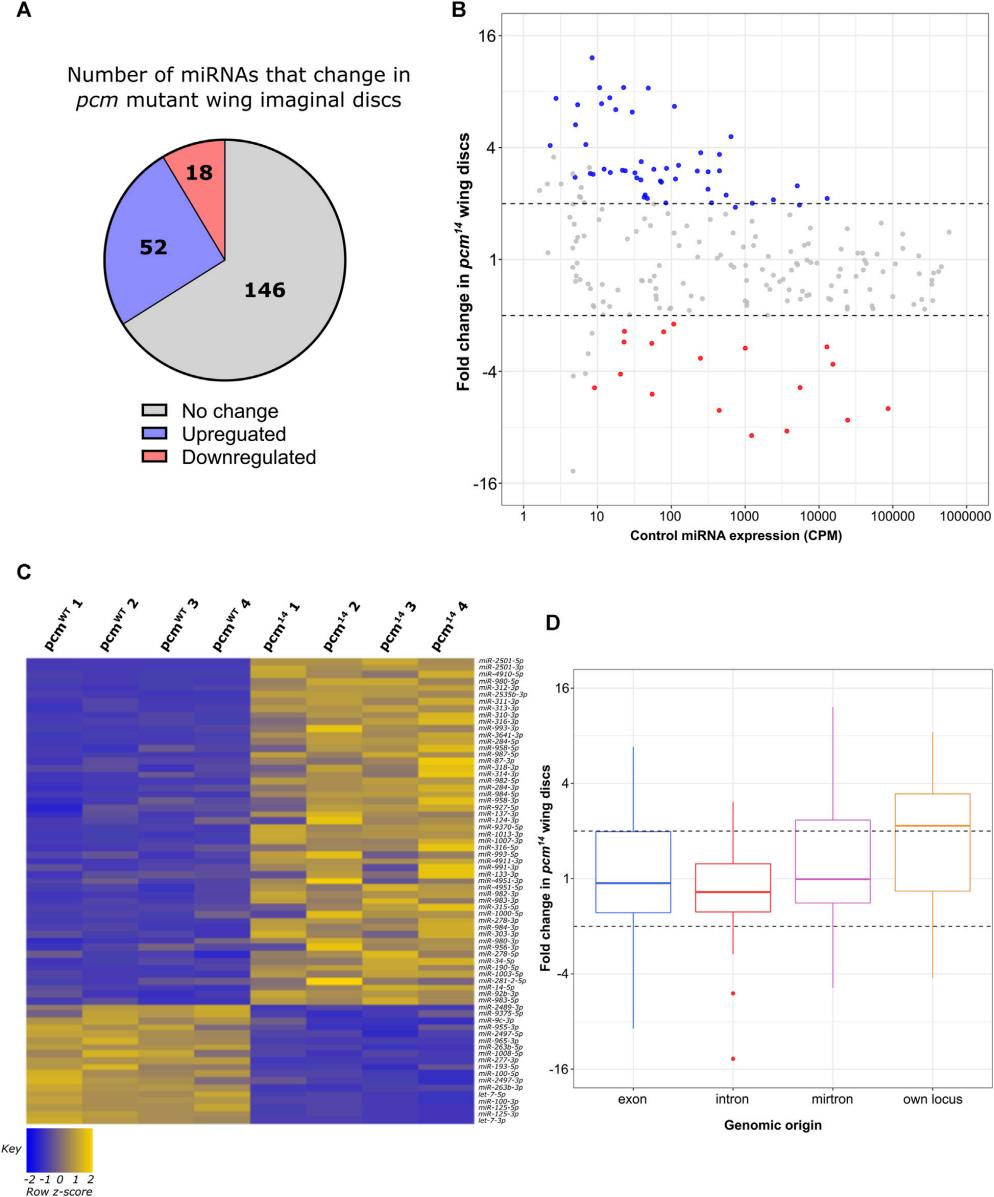
Loss of *pcm* results in changes in the miRNA landscape. **(A)** 30.8% of all miRNAs display significant differential expression in *pcm* ^*14*^ wing imaginal discs. miRNAs were determined as differentially expressed if a fold change is > 1.5 and called significant by both analysis pipelines. **(B)** MA plot displaying all miRNAs expressed in wing imaginal discs with upregulated miRNAs in blue and downregulated in red. Dotted lines represent ^±^2-fold change. Displayed CPM and fold change calculated using DESeq2. **(C)** Heatmap representing the expression of differentially expressed miRNAs in all replicates of *pcm* ^*WT*^ and *pcm* ^*14*^ wing imaginal discs. **(D)** Boxplot displaying fold change of all miRNAs in *pcm* ^*14*^ wing discs by genomic origin. Dotted lines represent ^±^2-fold change.

Having observed a limited effect of loss of Dis3L2 on the miRNA profile, we next sought to determine the extent that Pacman regulates the miRNA landscape, and to gain insights into the ways in which these miRNAs may induce the phenotypes observed. We again used the above miRNA-seq experiment to compare the levels of miRNAs between *pcm* ^*14*^ mutants and their isogenic controls (*pcm* ^*WT*^) using the same analysis pipeline. In contrast to our observations in *dis3L2* mutants, loss of Pacman resulted in the altered expression of 32% (70) of expressed miRNAs, with 80% of these demonstrating stabilisation in *pcm* ^*14*^ null tissues (Figures 3A–C). Interestingly, in contrast to *dis3L2* mutants, we observed a global trend of miRNAs transcribed from their own genomic locus displaying sensitivity to Pacman resulting in an enrichment of these miRNAs in the upregulated pool (Figure 3D; Supplementary Figures S2B–D). As with our analysis of Dis3L2-sensitive miRNAs, those miRNAs displaying Pacman sensitivity which were derived from host transcripts, largely showed regulation independent to that of their host (Supplementary Figure S2F).

### Select mirtrons and the *miR-310/13* cluster are particularly sensitive to Pacman

**FIGURE 4:**
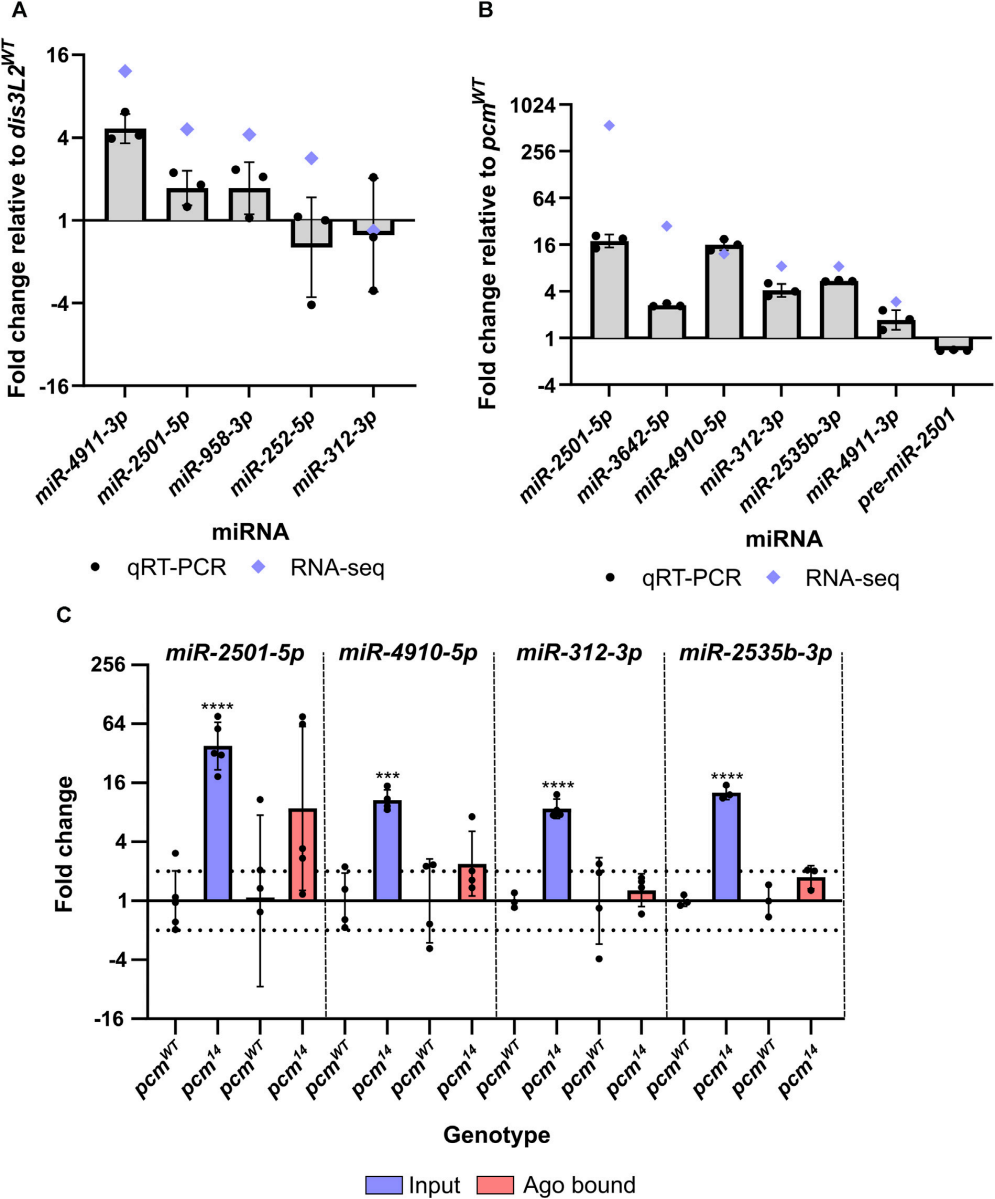
miRNAs differentially expressed in *pcm^14^* wing imaginal discs are not RISC-associated. **(A)** qRT-PCR validation of differentially expressed miRNAs in *dis3L2* ^*12*^ mutant tissues. Blue diamonds represent fold change by miRNA-seq. *n* = 3, error bars represent SD, statistical information can be found in Supplementary Table S1. **(B)** qRT-PCR validation of differentially expressed miRNAs in *pcm* ^*14*^ mutant tissues. Blue diamonds represent fold change by miRNA-seq. *n* = 3, error bars represent SD, statistical information can be found in Supplementary Table S2. **(C)** Assessment of RISC associated miRNAs. RNA from wing imaginal discs was isolated using the TraPR kit (Lexogen) and the differential expression between control and *pcm* ^*14*^ was assessed for input and Ago bound fractions. Differential expression was only identified in the input fraction suggesting the excess miRNA is not RISC associated. n ≥ 3, error bars represent SD, ***p < 0.001, ****p < 0.0001, no stars = p> 0.05. Full statistical information can be found in Supplementary Table S3.

As described above, five miRNAs which showed consistency between replicates were selected for verification by TaqMan qRT-PCR. This analysis showed that *miR-2501-5p*, *miR-3642-5p*, *miR-4910-5p*, *miR-312-3p* and *miR-2535b-3p* significantly increased in levels between 4-fold and 18-fold in the *pcm* ^*14*^ mutant compared to wild-type suggesting confidence in this dataset. We also validated the significant increase in *miR-4911-3p* in *dis3L2* ^*12*^ tissues (Figures 4A,B). *miR-2501-5p* was the most upregulated miRNA in *pcm* ^*14*^ tissues, and interestingly has previously been shown to be a tailed mirtron (Flynt et al., 2010) processed from an intron in its host gene *nulp1*. Therefore, we assessed the levels of its pre-miRNA in *pcm* ^*14*^ tissues. This analysis showed no change in expression (Figure 4B), consistent with our previous data that demonstrated no change in expression of *nulp1* mRNA in either *pcm* or *dis3L2* mutant tissues (Jones et al., 2016; Towler et al., 2020). This data therefore suggests that the increase in *miR-2501-5p* and *miR-2501-3p* is driven by defective post-transcriptional regulation in the absence of Pacman. Strikingly, the top 3 as well as 5 of the top 12 upregulated miRNAs are of the mirtron class suggesting a subset of mirtrons are particularly sensitive to Pacman activity.

Our data also show that *miR-312-5p, miR-312-3p miR-311-3p, miR-313-3p* and *miR-310-3p* are upregulated between 7-fold and 15-fold in the *pcm* mutant. All of these miRNAs are in the *miR-310/13* cluster and are encoded on separate transcripts next to each other on the 2^nd^ chromosome (Consortium et al., 2018). All four members of this *miR-310/13* cluster have been experimentally shown to regulate the Toll-mediated immune response via inhibiting the expression of Drosomycin in flies infected by bacteria (Li et al., 2017). Since all the detected miRNAs within this cluster are upregulated, we hypothesise that these changes are driven by transcriptional activation of the locus in response to a loss of Pacman, rather than a direct regulation by Pacman.

### Pacman sensitive miRNAs do not appear to be RISC-associated

Having observed a strong impact of Pacman mutations on the miRNA landscape we decided to determine whether selected upregulated miRNAs were likely to be functional (i.e., Ago bound within RISC), or if they were in a RISC-free miRNA pool awaiting degradation. To determine if the increased proportion of the miRNAs were functionally associated with RISC, we isolated RISC-miRNA complexes from *pcm* ^*WT*^ and *pcm* ^*14*^ wing imaginal discs using the TraPR kit from Lexogen. This allows the identification of small RNAs associated with a RISC using ion exchange chromatography. Free RNA stays bound on the positively charged column while small RNAs protected by RISC flow through and therefore RISC-associated miRNAs can subsequently be measured by TaqMan qRT-PCR. We validated the specificity of the kit for miRNAs by comparing the binding of a snoRNA, *snoR442*, with the selected miRNAs. Whilst all selected miRNAs were reliably detected by TaqMan qRT-PCR, *snoR442* was not. We next compared the expression of our selected Pacman-sensitive miRNAs in the input and RISC-associated fractions and whilst the input fraction displayed the expected change in expression in *pcm* ^*14*^ tissues, we did not observe a significant increase in the amount of miRNA associated with RISC (Figure 4C). This suggests that Pacman may degrade miRNAs that are either not loaded into a mature RISC or have been released from the RISC to await degradation.

### The developmentally important and conserved *let-7* cluster is Pacman-sensitive

**FIGURE 5:**
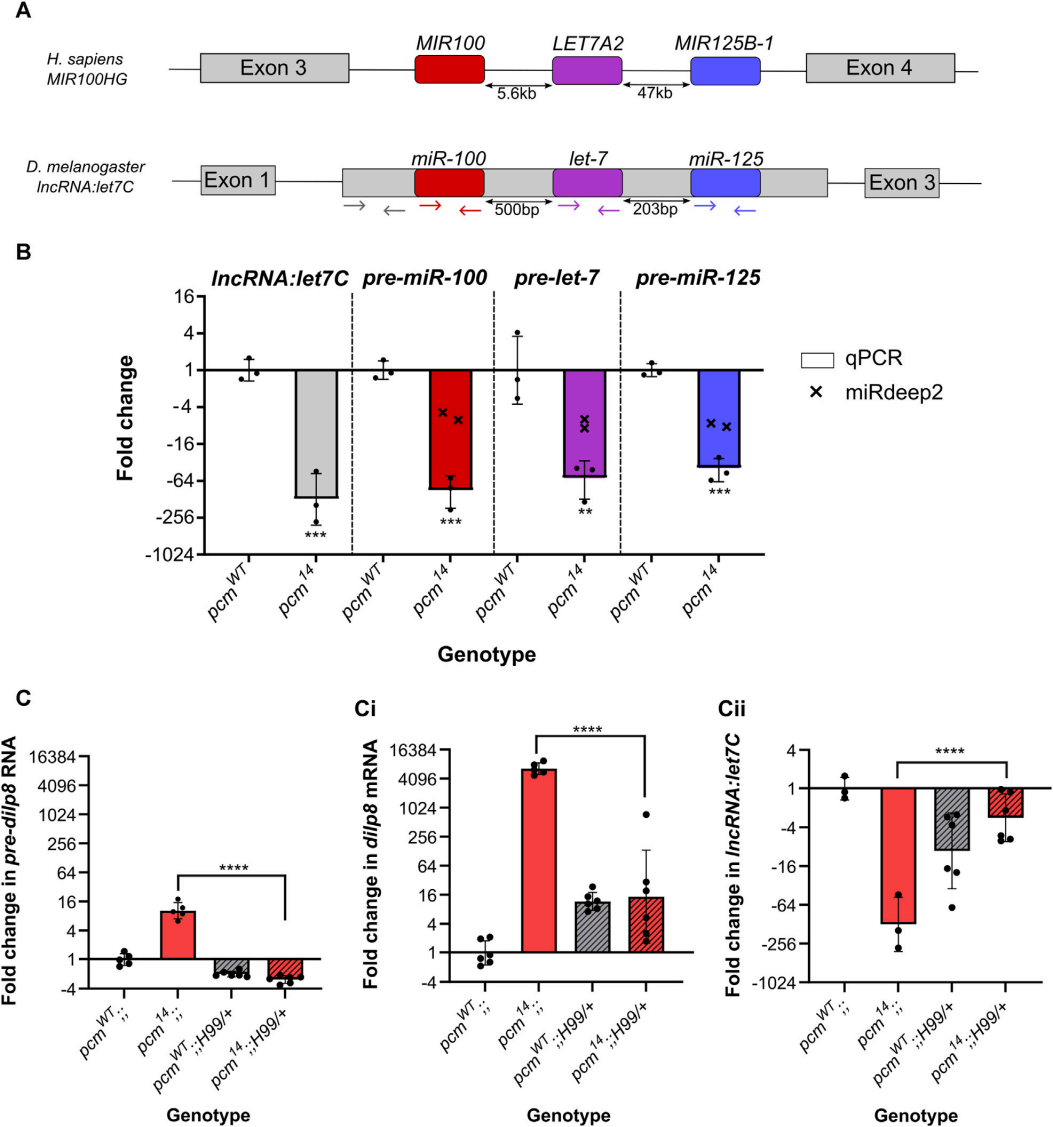
Increased apoptosis in *pcm^14^* wing discs drives the changes in expression of the *let7C* cluster and *dilp8*. **(A)** Genomic organisation of *let7C* demonstrating its conservation between humans and *Drosophila melanogaster*. Coloured arrows represent the location of the primers used in **(B)**. **(B)** The *let7C* host lncRNA and pre-miRNAs within the host are downregulated in *pcm* ^*14*^ wing discs. miRDeep2 data (crosses) display the fold change in both arms (5p and 3p) of the mature miRNAs by miRNA-seq. *n* = 3, error bars represent SD, **p < 0.01, ***p < 0.001. **(C)** The differential expression of *pre-dilp8* (i), *dilp8* (ii) and *lncRNA:let7C* (iii) in *pcm* ^*14*^ wing discs is largely driven by apoptosis as inhibition of apoptosis (using *H99*) in *pcm* ^*14*^ tissues rescues the altered expression. n ≥ 3, error bars represent SD, ****p < 0.0001. Statistical information for all comparisons is shown in Supplementary Table S4.

Given that the selected miRNAs with increased expression do not appear to be RISC-associated, we decided to ask if miRNAs demonstrating reduced expression may contribute to the Pacman loss of function apoptotic phenotype. We observed 18 miRNAs that displayed reduced expression in the absence of Pacman. Strikingly, 6 of these are located in the single, conserved *lncRNA:let7C.* This single lncRNA is the host of *miR-100, let-7* and *miR-125* (Sokol et al., 2008; Emmrich et al., 2014) with their downregulation in *pcm* ^*14*^ mutants ranging from −4.9-fold (*miR-100-5p*) to −8.9-fold (*let-7-3p*)*.* The *let7C* lncRNA miRNA cluster is conserved between flies and humans with the relative order of miRNAs also conserved (Sokol et al., 2008; Emmrich et al., 2014). In *Drosophila*, these miRNAs are located within the single exon of their host lncRNA, whilst in humans they are located in intron 3 (Figure 5A).

In *Drosophila*, null mutations in *let7C* result in lethality with a 43% penetrance, with surviving adults having defects in flight, mobility and fertility (Sokol et al., 2008). Studies on the phenotypic effects of the individual miRNAs within this complex showed that *let-7* is critical for the timing of cell cycle exit in wing imaginal discs (Reinhart et al., 2000; Moss, 2007) as well as other phenotypes including severe defects in female fertility and oviposition (Sokol et al., 2008). We therefore decided to further investigate the effect of Pacman depletion on *let7C* biology. First, we validated the change in each pre-miRNA hairpin by qRT-PCR and showed a strong downregulation (Figure 5B) explaining the downregulation of both arms (5p- and 3p-) in our miRNA-seq data. Next, we asked if this was a result of downregulation of the host transcript, *lncRNA:let7C*, or if it was a dysregulation of pre-miRNA processing, again using qRT-PCR. Using primers specific for the lncRNA (Figure 5A) we demonstrated a strong reduction in *lncRNA:let7C*, consistent with that of the pre-miRNAs suggesting that this was indeed driven by a downregulation of the host lncRNA, likely driven by a reduction in transcription (Figure 5B).

One of the most studied targets of *let-7* and *miR-125* is *chronologically inappropriate morphogenesis* (*chinmo*) (Wu et al., 2012; Chawla et al., 2016; Pandey et al., 2021) which acts in a dosage-dependent manner to regulate the temporal identity and viability of neurones (Zhu et al., 2006; Chawla et al., 2016). To ask if the downregulation of *let-7* and *miR-125* is functional, we assessed *chinmo* expression in *pcm* mutant tissues and observed a significant increase in our previous RNA-seq data (Jones et al., 2016), which we validated as a 23.1-fold increase by qRT-PCR (Supplementary Figure S3). This therefore suggests that the reduction in *chinmo* required for a timely developmental transition is absent in *pacman* mutants as a result of decreased *let-7* and *miR-125* expression.

To further probe this pathway, we turned to our previous work, in which we showed that the insulin-like peptide Dilp8, which co-ordinates tissue growth with developmental timing, is upregulated in *pcm* mutants at the transcriptional and post-transcriptional levels (Jones et al., 2016). Dilp8 has previously been reported to be secreted from the imaginal discs in vesicle-like structures in response to tissue damage (Colombani et al., 2012; Garelli et al., 2012). This hormone then travels through the haemolymph to remote tissues such as the brain to suppress ecdysone production (Garelli et al., 2012; Colombani et al., 2015; Garelli et al., 2015). Lower levels of ecdysone production delay development and the onset of pupariation (Garelli et al., 2012; Colombani et al., 2015; Garelli et al., 2015). In *Drosophila* Kc-167 and S2 cultured cells, dissected organs and pre-pupae, the steroid hormone ecdysone (20-hydroxyecdysone) is required for expression of *let-7* and *let7C* (Sempere et al., 2002; Sempere et al., 2003; Chawla and Sokol, 2012) although other regulatory factors may also be involved (Bashirullah et al., 2003; Luhur et al., 2014). The effect of Pacman on *dilp8* transcripts is specific, as *dilp6*, the only other member of the Dilp family we detect in wing imaginal discs, is unchanged at the RNA level in mutant discs compared with controls (Jones et al., 2016).

**FIGURE 6:**
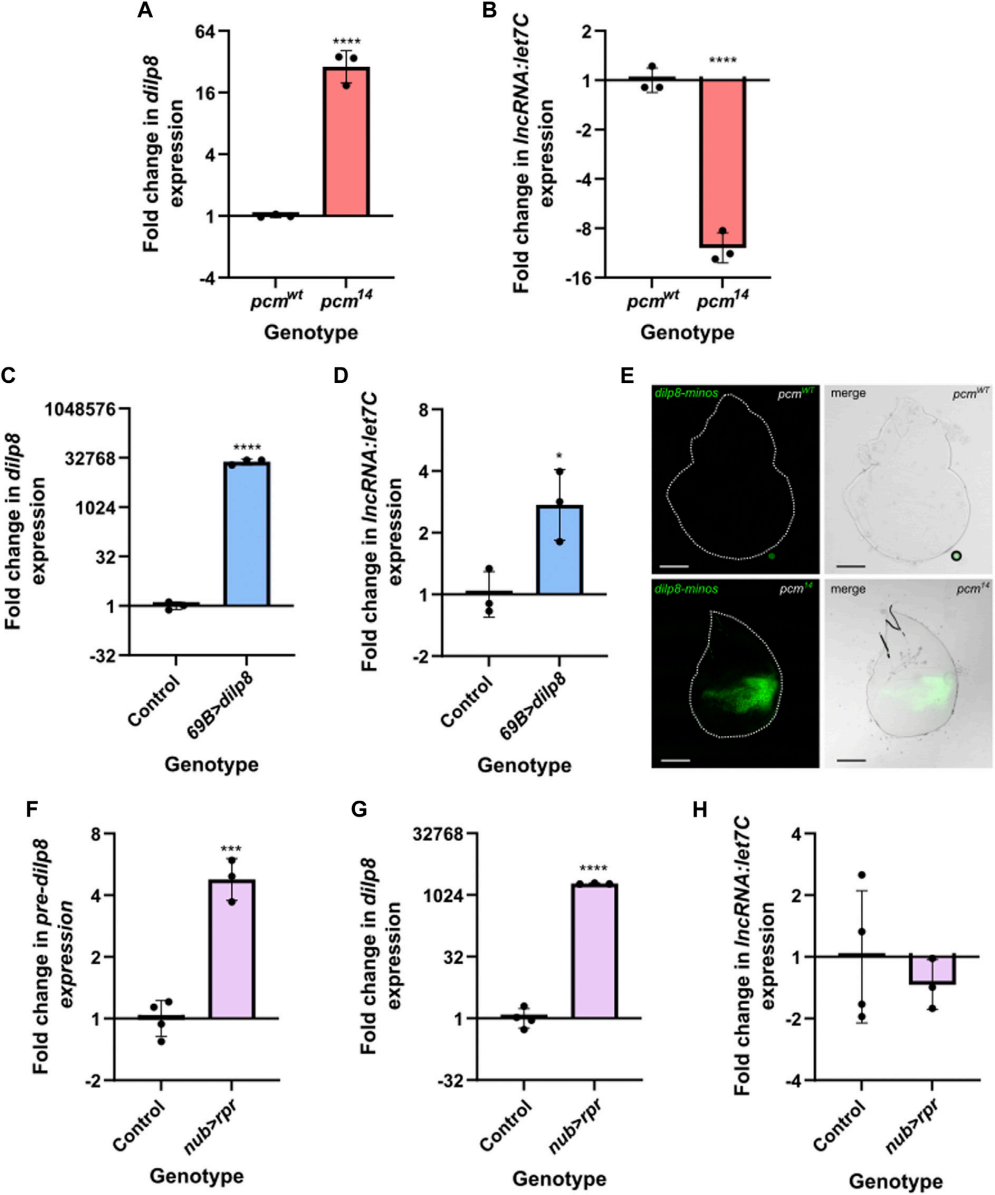
The induction of apoptosis or increased *dilp8* expression alone is insufficient to drive downregulation of *let7C*. **(A–B)** Increased expression of *dilp8* **(A)** and decreased expression of *let7C* **(B)** can be observed in *pcm* ^*14*^ L3 larvae compared to controls. *n* = 3 error bars represent SD, ****p ≤ 0.0001. **(C–D)** Overexpression of *dilp8* throughout the wing imaginal disc using *69B-GAL4* is not sufficient to drive *let7C* downregulation. **(C)** Demonstrates *dilp8* expression and **(D)** demonstrates *let7C* expression in wing imaginal discs. *n* = 3, error bars represent SD, ****p < 0.0001, *p ≤ 0.05. **(E)** Increased expression of *dilp8-minos* reporter in *pcm* ^*14*^ mutant wing imaginal discs compared to *pcm* ^*WT*^ control. *n* ≥ 15, scale bar represents 100 µm. **(F–H)** Driving *UAS-rpr* in the wing pouch of the wing imaginal disc with *nub-GAL4* to induce apoptosis is sufficient to cause increased *pre-dilp8* **(F)** and *dilp8* **(G)** expression but not the downregulation of *let7C* **(H)**. *n* ≥ 3, error bars represent SEM, ***p < 0.001, ****p < 0.0001. For all statistical information see Supplementary Table S5.

Therefore, an attractive hypothesis is that the loss of Pacman results in upregulation of Dilp8 (as previously shown (Jones et al., 2016)), resulting in lower levels of ecdysone in the haemolymph and reduced expression of *let7C* and its encoded miRNAs *miR-100*, *miR-125* and *let-7*. Since the involvement of Dilp8 in *let7C* regulation has not yet been tested, we decided to probe the potential relationship. We first confirmed the systemic increase in *dilp8* and reduction in *let7C* expression throughout *pcm* ^*14*^ larvae (Figures 6A, B), and subsequently used the *GAL4-UAS* system to ectopically express wild-type *dilp8* throughout the wing disc (*69B-GAL4*). Using TaqMan qRT-PCR, we demonstrated successful ectopic expression of *dilp8* RNA (Figure 6C), however, this resulted in a slight increase in *let7C* expression rather than the reduction observed in *pcm* ^*14*^ tissues (Figure 6D). Therefore, the increase in *dilp8* expression alone is not sufficient to drive the observed reduction in *let7C.*

Our previous work used qRT-PCR to assess the transcriptional contribution towards the increase of *dilp8* expression in *pcm* mutant wing imaginal discs. This approach assessed changes in the levels of mature *dilp8* vs. those for *pre-dilp8*. This analysis demonstrated a stronger increase in mature *dilp8* (6600-fold), however, we did observe a 10.3-fold increase in *pre-dilp8* suggesting a transcriptional contribution. (Jones et al., 2016). Taking this data together with previous work showing that *dilp8* is secreted in response to tissue damage (Colombani et al., 2012; Garelli et al., 2012), we hypothesised that the apoptosis induced by loss of Pacman (Waldron et al., 2015) may contribute to the observed changes in *dilp8* and *let7C.* To address this, we inhibited apoptosis using a heterozygous *H99* deletion which removes a single copy of the pro-apoptotic genes *hid, grim* and *rpr* (Waldron et al., 2015). When used as a heterozygote in *pcm* ^*14*^ mutants (*pcm* ^*14*^ *;;Df(3L)H99/+*), the *H99* deletion rescues wing disc size from 45% to 81% compared to the wild type control. This rescue of wing disc size is greater than that achieved using *UAS-DIAP* in a *pcm* ^*14*^ mutant background (*pcm* ^*14*^; *GAL80* ^*ts*^ */+; 69B-GAL4/UAS-DIAP:* 45%–67%) and similar to that achieved when another pro-apoptotic gene (*Ark*) is mutated, which justifies the use of the *H99* deletion. Inhibiting apoptosis using the *H99/+* deletion in a *pcm* mutant (*pcm* ^*14*^ *;;Df(3L)H99/+*) reduced Caspase 3 staining and also rescued the delay in wing imaginal disc development as determined by Wingless staining (Waldron et al., 2015).

In *pcm* ^14^ mutants carrying the *H99* deletion (*pcm* ^*14*^ *;;Df(3L)H99/+*), where little to no apoptosis is taking place (Waldron et al., 2015), the levels of *pre-dilp8*, *dilp8* and *let7C* were not statistically different from wild-type controls carrying the *H99* deletion *pcm* ^*WT*^ *;;Df(3L)H99/+* demonstrating the apoptosis induced by loss of Pacman is responsible for their changes (Figure 5C). To directly assess the transcriptional contribution to our observations, we made use of a GFP enhancer trap line to provide a genetic readout of *dilp8* transcription. Whilst we see minimal GFP expression in a *pcm* ^*WT*^ background, we observed a striking increase in GFP expression in *pcm* ^*14*^ tissues confirming a significant transcriptional contribution towards the increase in *dilp8* expression (Figure 6E).

Given our observation of a transcriptional contribution to increased *dilp8* expression in *pcm* mutants and the rescue of *dilp8* expression in *H99* mutants, we next hypothesised that driving apoptosis alone, to mimic that caused by loss of Pacman, may be sufficient to increase *dilp8* expression. Therefore, to determine whether apoptosis alone is sufficient for the observed changes in *dilp8* and *let7C* expression, or if Pacman-induced apoptosis is specifically required, we used the *nubbin-GAL4 driver* to ectopically express the pro-apoptotic gene *rpr* specifically in the wing pouch of the wing imaginal disc. This resulted in an increase in *pre-dilp8* (and a substantial increase in mature *dilp8* expression ((Figures 6F–G)), however, these were 3-fold less that that observed in *pcm* mutants suggesting that whilst apoptosis alone drives increased *dilp8* expression, loss of Pacman may accentuate these effects potentially due to defects in post-transcriptional regulation. Despite the increase in *pre-dilp8* and *dilp8,* we did not observe a reduction in *let7C* expression (Figure 6H). Therefore, these data suggest that whilst apoptosis alone is sufficient to drive changes in *dilp8* expression, the loss of Pacman *and* apoptosis are responsible for the reduction in *let7C* expression. This demonstrates a crucial role for Pacman in regulating these developmentally important miRNAs.

**FIGURE 7:**
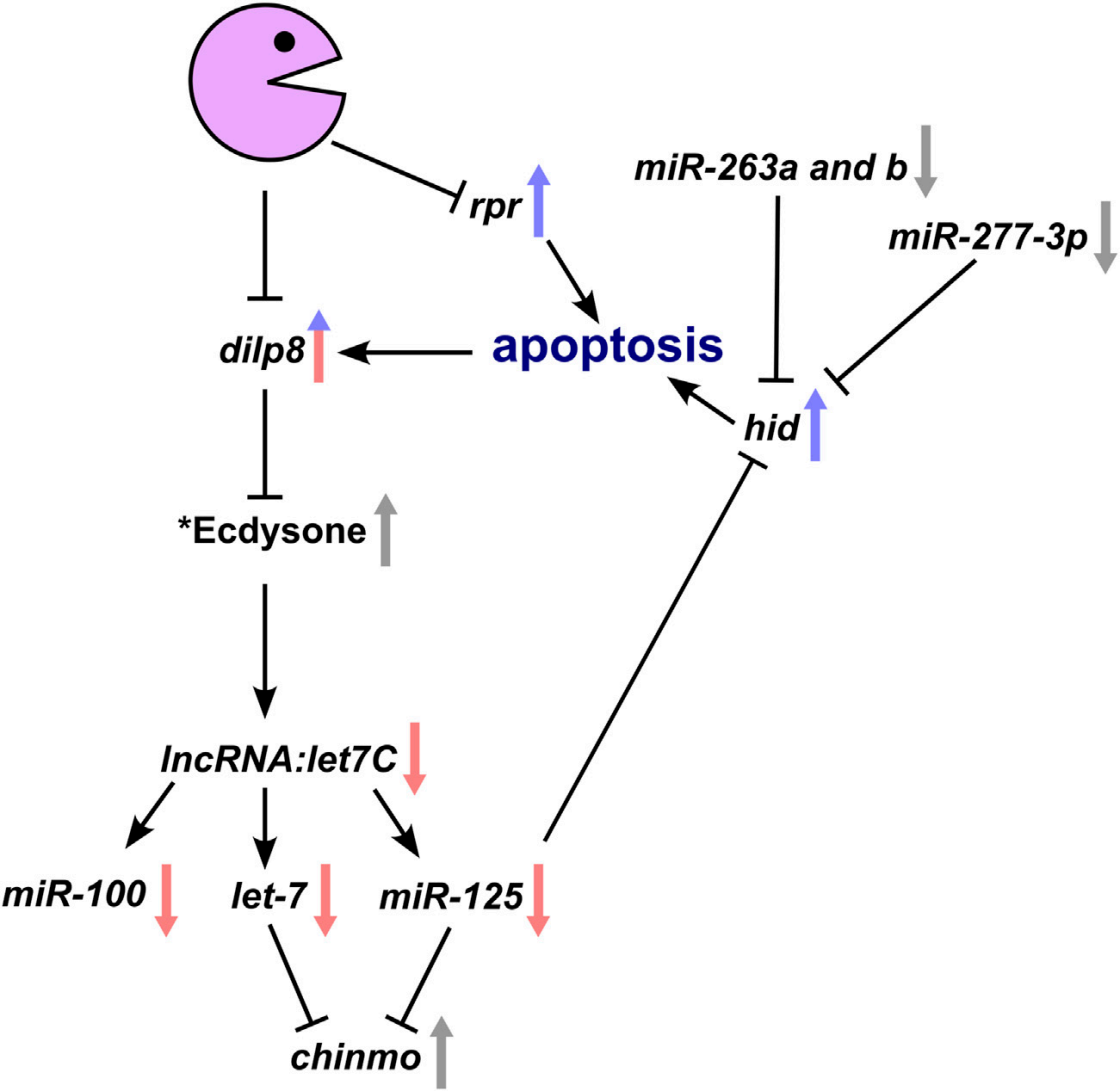
Genetic model whereby loss of Pacman results in the observed changes in gene expression and subsequent apoptotic phenotype. Interactions within the model have all been experimentally verified either by our group or by others. Arrows demonstrate the direction of change in a *pacman* mutant with red and blue arrows representing transcriptional and post-transcriptional changes respectively. Grey arrows represent verified changes for which the transcriptional and/or post-transcriptional contribution is yet to be determined. *Whilst the effect of Dilp8 on ecdysone has been shown, the hypothesised reduction in ecdysone in *pacman* mutants remains to be determined as does the Pacman-induced apoptosis specific mechanism inhibiting *let7C* expression.

Confirmation that Pacman regulates a complex network of proteins and RNAs which normally prevent apoptosis is supported by further inspection of our miRNA-seq data. As can be seen in Figure 3C and Supplemental File S1, *miR-277*, *miR-263a* and *miR-263b* are also downregulated in *pcm* ^*14*^ wing imaginal discs. It has previously been shown that a decrease in *miR-125, 277* and *263a* and *263b* results in increased *hid* expression because *hid* includes binding sites for these RNAs in its 3′UTR (Hilgers et al., 2010; Bejarano et al., 2021). Our previous work (Waldron et al., 2015) showed that levels of the pro-apoptotic RNAs *reaper*, *hid* and *grim* increase during apoptosis, with *reaper* and *hid* RNAs being upregulated at the post-transcriptional level in *pcm* null mutants (Waldron et al., 2015; Jones et al., 2016). These results are entirely consistent with a positive feedback pathway that occurs in *pacman* mutants to increase apoptosis (Figure 7).

Our data therefore show the importance of Pacman in regulating miRNA levels in naturally developing tissues and provide insights into the ways that ribonucleases can integrate their effects into developmentally important pathways.

## Discussion

In this study, we have used miRNA-seq in a global approach to identify candidate targets of the exoribonucleases Dis3L2 and Pacman during wing imaginal disc development. The use of well-characterised null mutations plus their isogenic controls has allowed us to assess the effects of these exoribonucleases on mature miRNA expression as well as reveal insights into the global expression of miRNAs in a natural tissue. Our studies also allowed us to increase our understanding of the contribution of Pacman in inhibiting apoptosis in wing imaginal discs via the *let-7* miRNA complex. This work shows that cytoplasmic 5′-3′ RNA degradation plays a pivotal role in maintenance of the correct levels of miRNAs to regulate a suite of RNAs involved in this important cellular event.

Analysis of our miRNA-seq data revealed, for the first time, the global landscape of miRNA expression in relation to their genomic location in the wing imaginal disc. Interestingly, most detected miRNAs are located within mRNAs (54%), although a substantial proportion are located within lncRNAs (27%). Of note, miRNAs encoded within lncRNAs, together with their host lncRNAs, tend to be more highly expressed than other miRNAs with their host lncRNAs also being longer and more abundant than average. The molecular mechanisms underlying these effects are not known. This data also shows that mirtrons are often less well expressed than other miRNAs, as previously observed (Berezikov et al., 2011).

The 3′-5′ exoribonuclease Dis3L2 was shown to have a minimal effect on the miRNA expression profile, however, we did validate an increase in the expression of *miR-4911-3p.* Interestingly, the *miR-4911* pre-miRNA is encoded within the second intron of the gene *ND23,* and therefore belongs in the category of mirtrons. A number of mirtrons have been shown in *Drosophila* to be preferentially uridylated by the TUTase Tailor and subsequently degraded by Dis3L2 (Bortolamiol-Becet et al., 2015; Reimao-Pinto et al., 2015). Tailor preferentially uridylates transcripts ending in a guanine (which is the 3′-most nucleotide following splicing), which is indeed the case for *miR-4911-3p* and the addition of uridyl residues to the G-terminal mirtrons produces a preferential substrate for Dis3L2, therefore our findings are entirely consistent with a role for Dis3L2 in degradation of this mirtron-derived miRNA.

Previous work in *Drosophila* S2 cells revealed that the depletion of Tailor results in an increase of a substantial number of miRNAs (35%; 28 out of the 79 detected) (Reimao-Pinto et al., 2015). If uridylation by Tailor occurs at the same levels in wing imaginal discs, and the uridylated transcripts are predominantly degraded by Dis3L2, then we would expect to see upregulation of more miRNAs in *dis3L2* mutant imaginal discs. However, we see very few changes in miRNA expression which may suggest the wing imaginal disc is either less dependent upon this pathway for miRNA decay, or, more plausibly, there is redundancy between the miRNA decay pathways. For example, our previous work showed that loss of Dis3, another 3′-5′ exoribonuclease, which associates with the exosome, resulted in the altered expression of 39% of miRNAs in the wing imaginal disc (42 of 109 detected) (Towler et al., 2015). Therefore, although Dis3L2 affects the levels of pre-miRNAs, as has been shown in mouse and human cells, the levels of functional mature miRNAs appear to be less dependent upon Dis3L2 mediated decay. This is also consistent with other findings (Chang et al., 2013; Ustianenko et al., 2013; Nowak et al., 2017), although a more pronounced role was identified in immortalised human cells which also demonstrated the importance of 3′ terminal modification within the process (Shukla et al., 2019).

In contrast, loss of the 5′-3′ exoribonuclease Pacman has a much more profound effect on the levels of miRNAs in wing imaginal discs with 70 (32% of the total detected) being dysregulated, the majority of which (52) were upregulated. These results are consistent with those in *C. elegans* where depletion of Xrn-1 resulted in accumulation of most of the miRNAs tested, including *let-7* (Chatterjee et al., 2011). In humans, XRN1 has been shown to regulate a subset of miRNAs in immortalised cell lines (HEK293T (Bail et al., 2010)) although the contribution of XRN1 in normal human tissue has not yet been tested. miRNAs transcribed from their own locus showed a trend of increased expression in the absence of Pacman. Interestingly, among the top 12 upregulated miRNAs, 5 (41.67%) were mirtrons suggesting that Pacman may be key in regulating the levels of these non-canonical miRNAs. Our studies also increase our understanding of the contribution of Pacman to apoptosis in wing imaginal discs and its subsequent effect on the *let-7* miRNA cluster. This work shows that cytoplasmic 5′-3′ RNA degradation plays a pivotal role in maintenance of the correct levels of miRNAs to regulate a suite of RNAs involved in developmental timing and maintaining cellular homeostasis.

The biological function of the top 15 upregulated miRNAs is unknown, except for *miR-310*, *miR-311*, *miR-312* and *miR-313*, which are expressed as a cluster. These have been experimentally shown to co-target the *drosomycin* 3′UTR and regulate the Toll-mediated immune response (Li et al., 2017) as well as influencing normal synaptic homeostasis (Tsurudome et al., 2010) and acting as an antagonist to β-catenin (*armadillo*) function during differentiation of testes cells in *Drosophila* (Pancratov et al., 2013). Interestingly, the null mutation in *pacman* appears to result in upregulation of immune response genes which include mRNAs encoding proteins such as CecropinC (CecC), IM33 (CG16712), AttacinA (AttA) and Nplp2 (Jones et al., 2016) as well as the *miR-310/13* cluster. The signalling pathway(s) regulated by Pacman to activate these immune response genes are unclear. However, this “sterile inflammation” has previously been associated with tissue damage, wound healing and cell competition which involves upregulation of the JNK pathway (Shaukat et al., 2015; Byun et al., 2019). Since our previous work showed that *pacman* genetically interacts with the JNK pathway (Grima et al., 2008) it is possible that this is the signalling pathway indirectly regulated by Pacman to induce the immune response genes. This suggestion is supported by data showing that JNK signalling can also stimulate *dilp8* expression (Demay et al., 2014). Another possibility is that Pacman indirectly modulates the Hippo pathway as the transcriptional activators Yorkie and Scalloped have been shown to directly regulate *dilp8* expression (Boone et al., 2016).

The most significant finding in this paper is that Pacman controls the expression of *lncRNA*:*let7C,* encoding the conserved miRNAs *miR-100*, *let-7* and *miR-125* which in turn regulate development and apoptosis. The data in this publication explores the hypothesis that loss of Pacman results in Dilp8 upregulation, which suppresses ecdysone production, causing a decrease in *let7C* levels through an as of yet undetermined factor (Figure 7). By driving expression of the pro-apoptotic RNA *rpr*, we showed that apoptosis alone was sufficient to increase levels of *pre-dilp8*, showing a transcriptional effect which must be an indirect effect of Pacman. However, the increase of *dilp8* mRNA showed a much larger fold change (1024-fold compared to 5-fold) suggesting that *dilp8* is also post-transcriptionally upregulated during apoptosis, as shown in our previous publication (Jones et al., 2016). Our current hypothesis, which incorporates ideas from other work (Blanco-Obregon et al., 2022) is that the apoptosis observed in Pacman mutants drives an increase in Dilp8, potentially though the JNK and/or Hippo pathways, and that Pacman may normally function to temper this increase by degrading excess *dilp8* RNA. Therefore, Pacman may regulate Dilp8 transcriptionally (i.e., indirectly) and post-transcriptionally (i.e., directly) during apoptosis to produce a “spike” in expression of Dilp8, allowing wing disc growth to react quickly to developmental and environmental conditions. In contrast, induction of apoptosis alone did not result in a reduction of *let7C* levels, suggesting that the loss of Pacman and apoptosis are required for the observed decrease in *let7C* expression with further work required to unlock this mechanism.

Interestingly, *miR-277*, *263a* and *263b*, which target the 3′UTR of the pro-apoptotic RNA *hid*, are also downregulated in *pcm* null mutants. In our previous work (Waldron et al., 2015), we showed that *reaper* and *hid* are regulated post-transcriptionally by Pacman. Therefore, the data in this paper, together with our previous work, allows us to construct a model which helps to explain the effects of Pacman on apoptosis in wing imaginal discs (Figure 7).

In summary, our experiments, using natural tissue rather than immortalised cell lines, show that the 5′-3′ exoribonuclease Pacman/XRN1 plays an important role in controlling the levels of mature miRNAs. Pacman also regulates the levels of mRNAs, lncRNAs and miRNAs (directly and indirectly) in the apoptosis pathway, linking the molecular effects on RNA levels with the Pacman phenotypes observed. Further studies should reveal the molecular mechanisms whereby this exoribonuclease specifically controls the levels of RNAs in wing imaginal discs to allow correct development and maintain homeostasis.

## Data Availability

The original contributions presented in the study are publicly available. Raw RNA sequencing files have been deposited in ArrayExpress (https://www.ebi.ac.uk/arrayexpress/). Accession number: E-MTAB-13781.
