# Supplementary material for: Characterisation of the in-vivo miRNA landscape in Drosophila ribonuclease mutants reveals Pacman-mediated regulation of the highly conserved let-7 cluster during apoptotic processes
Source: Front Genet. 2024 Feb 20;15:1272689. doi: 10.3389/fgene.2024.1272689 (PMC10912645; doi:10.3389/fgene.2024.1272689)
Supplement: Supplementary file 1 [file DataSheet1.zip › Supplemental_Figure_Legends_and_Tables.pdf]

Supplemental Figure Legends

Supplemental Fig S1: Descriptive statistics of miRNAseq in wing imaginal discs. A) *dis3L2*^{WT} and *pcm*^{WT} miRNA expression show excellent correlations across all replicates. **B)** Correlation between the average normalised expression (CPM) of each detected miRNA between the two isogenic control lines demonstrates excellent correlation. **C)** miRNAs derived from host genes are globally more abundant than those transcribed from their own genomic locus. miRNA expression refers to the average CPM across all 8 isogenic control replicates. **D)** lncRNAs hosting miRNAs are longer than the genome average.

Supplemental Fig S2: miRNA vs host relationships in ribonuclease mutants. A/B) Boxplot displaying the fold change of miRNAs in *dis3L2*^{12} **(A)** or *pcm*^{14} **(B)** mutant wing discs by host RNA type. Dotted lines represent $^{\pm}$2 fold change. **C/D)** Proportion of miRNAs detected in wing discs and demonstrating ribonuclease sensitivity by genomic locus **(C)** or host RNA type **(D)**. A larger than expected proportion of miRNAs derived from their own locus show increased expression in ribonuclease mutants. **E/F)** Most changes in miRNA expression are independent of changes in their host RNA in either *dis3L2*^{12} **(E)** or *pcm*^{14} **(F)** mutant wing discs. Dotted lines represent $^{\pm}$2 fold change.

Supplemental Fig S3: *chinmo* expression is increased in *pcm* mutant wing imaginal discs. qRT-PCR validating increased *chinmo* expression in *pcm*^{14} wing imaginal discs. n=3, error bars represent SEM, ***$p<0.0002$.

Supplemental Tables

Table S1. Fold change and statistical significance of miRNAs dysregulated in *dis3L2*[12] WIDs presented in Figure 4A. The data was retrieved from qPCR experiments performed to verify miRNA levels and from the sRNA-seq data. padj= adjusted p-value. n=3 for qPCR and n=4 for sRNA-seq data.

	qRT-PCR		RNA-seq	
**miRNA**	**Fold change**	**p-value**	**Fold change**	**padj**
miR-4911-3p	3.55	0.0049	12.23	$9.49*10^{-8}$
miR-2501-5p	1.71	0.1188	4.34	$1.50*10^{-2}$
miR-958-3p	1.71	0.3646	4.18	$9.5*10^{-9}$
miR-252-5p	-1.57	0.4143	2.84	$1.19*10^{-8}$

Table S2. Fold change and statistical significance of miRNAs dysregulated in *pcm*[14] WIDs presented in Figure 4B. The data was retrieved from qPCR experiments performed to verify miRNA levels and from the sRNA-seq data. padj= adjusted p-value. n=3 for qPCR and n=4 for sRNA-seq data.

	qRT-PCR		RNA-seq	
**miRNA**	**Fold change**	**p-value**	**Fold change**	**padj**
miR-2501-5p	17.83	0.0007	544.36	$8.5*10^{-17}$
pre-miR-2501	1.44	0.0012	n/a	n/a
miR-3642-5p	2.66	0.026	27.67	$9.51*10^{-4}$
miR-4910-5p	15.94	0.0031	12.15	$3.59*10^{-22}$
miR-312-3p	4.12	0.0003	8.41	$6.21*10^{-19}$
miR-2535b-3p	5.46	<0.0001	8.34	$1.02*10^{-45}$

Table S3. Fold change and Statistical information for TRAPR experiments in Figure 4C. The data was retrieved from qPCR experiments performed to assess if the observed change in miRNA expression contributed to the amount of miRNA bound by Ago. n=3

	Input fraction		Ago bound fraction	
**miRNA**	**Fold change**	**p-value**	**Fold change**	**p-value**
miR-2501-5p	38.08	<0.0001	8.78	0.1195
miR-4910-5p	10.66	0.0005	2.39	0.2112
miR-312-3p	8.73	<0.0001	1.28	0.6622
miR-2535b-3p	12.70	<0.0001	1.75	0.1073

Table S4A-C. Fold change and statistical significance of *pre-dilp8* (A), *dilp8* (B) and *let-7-C* (C) in *pcm*^{WT} and *pcm*^{14} wing imaginal discs with and without apoptotic inhibition (*H99/+*) presented in Figure 5. p-value derived from ANOVAs, n=3.

A

Comparison	Fold change	p-value
pcm^{WT} vs *pcm*^{14}	10.3	<0.0001
pcm^{WT} vs *pcm*^{WT}*;;H99/+*	2.0	0.0016
pcm^{WT} vs *pcm*^{14}*;;H99/+*	2.6	<0.0001
pcm^{14} vs *pcm*^{WT}*;;H99/+*	20.6	<0.0001
pcm^{14} vs *pcm*^{14}*;;H99/+*	26.6	<0.0001
pcm^{WT}*;;H99/+* vs *pcm*^{14}*;;H99/+*	1.3	0.3399

B

Comparison	Fold change	p-value
pcm^{WT} vs *pcm*^{14}	6608.0	<0.0001
pcm^{WT} vs *pcm*^{WT}*;;H99/+*	11.7	0.0107
pcm^{WT} vs *pcm*^{14}*;;H99/+*	14.7	0.0052
pcm^{14} vs *pcm*^{WT}*;;H99/+*	565.0	<0.0001
pcm^{14} vs *pcm*^{14}*;;H99/+*	449.4	<0.0001
pcm^{WT}*;;H99/+* vs *pcm*^{14}*;;H99/+*	1.3	0.9872

C

Comparison	Fold change	p-value
pcm^{WT} vs *pcm*^{14}	128.6	0.0003
pcm^{WT} vs *pcm*^{WT}*;;H99/+*	9.3	0.0390
pcm^{WT} vs *pcm*^{14}*;;H99/+*	2.8	0.5030
pcm^{14} vs *pcm*^{WT}*;;H99/+*	13.9	0.0137
pcm^{14} vs *pcm*^{14}*;;H99/+*	45.2	0.0007
pcm^{WT}*;;H99/+* vs *pcm*^{14}*;;H99/+*	3.3	0.2432

Table S5. Fold change and p-value for statistical comparisons made in Figure 6. p-value derived from t-tests.

Panel number	Comparison	Fold change	p-value
**Ai**	*pcm*^{WT} vs *pcm*^{14} L3 larvae	28.56	<0.0001
**Aii**	*pcm*^{WT} vs *pcm*^{14} L3 larvae	-10.53	0.0001
**Bi**	Parental control vs *69B>dilp8* wing imaginal discs	24661	<0.0001
**Bii**	Parental control vs *69B>dilp8* wing imaginal discs	2.73	0.0208
**Ci**	Parental control vs *nub>rpr* wing imaginal discs	4.77	0.0002
**Cii**	Parental control *vs nub>rpr* wing imaginal discs	1951	<0.0001
**Ciii**	Parental control vs *nub>rpr* wing imaginal discs	-1.366	0.5279
