# Supplementary figures and images for: Characterisation of the in-vivo miRNA landscape in Drosophila ribonuclease mutants reveals Pacman-mediated regulation of the highly conserved let-7 cluster during apoptotic processes
Source: Front Genet. 2024 Feb 20;15:1272689. doi: 10.3389/fgene.2024.1272689 (PMC10912645; doi:10.3389/fgene.2024.1272689)

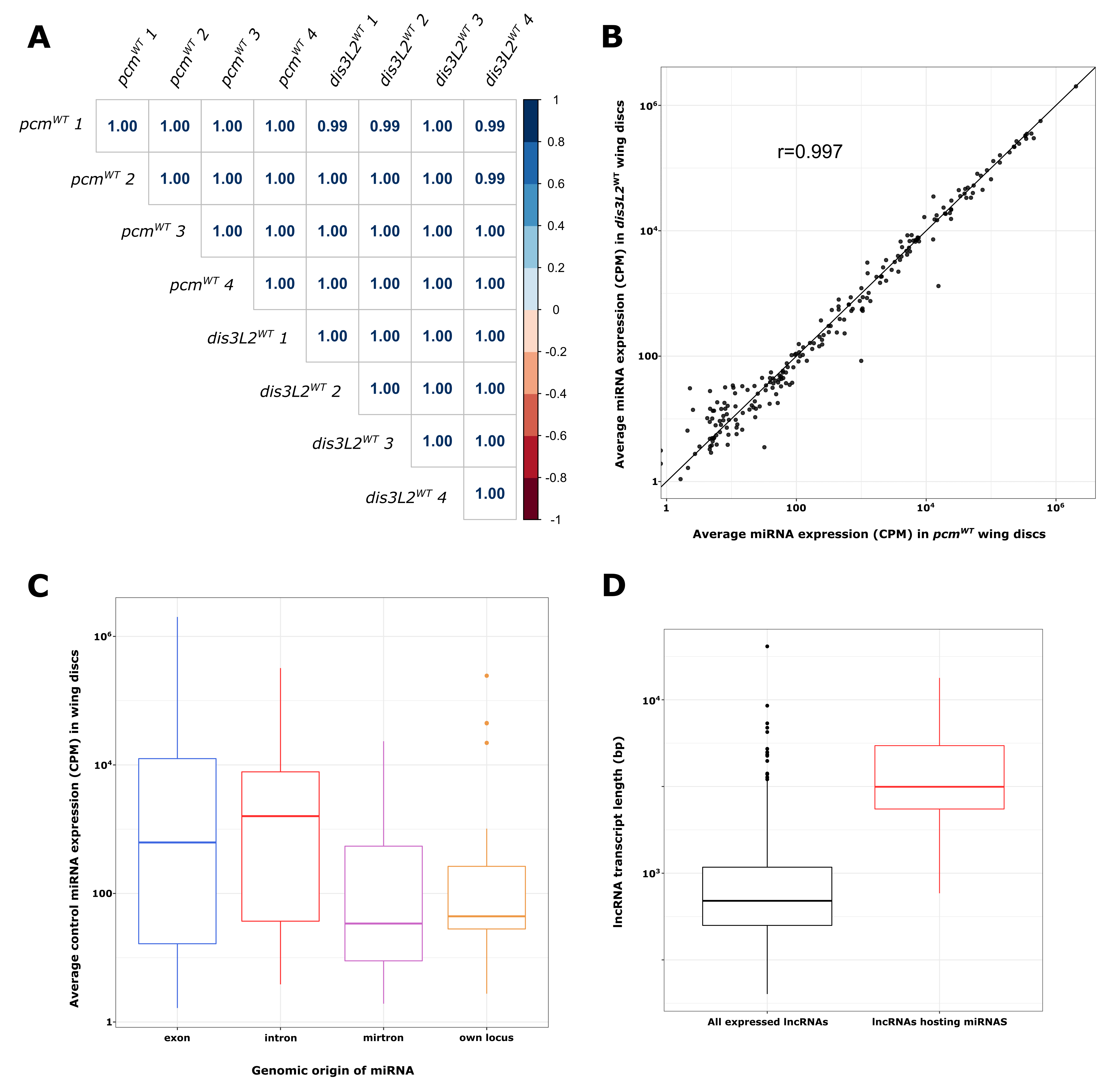

Supplement: Supplementary file 1 [file DataSheet1.zip › SupplementalFig1.tif]

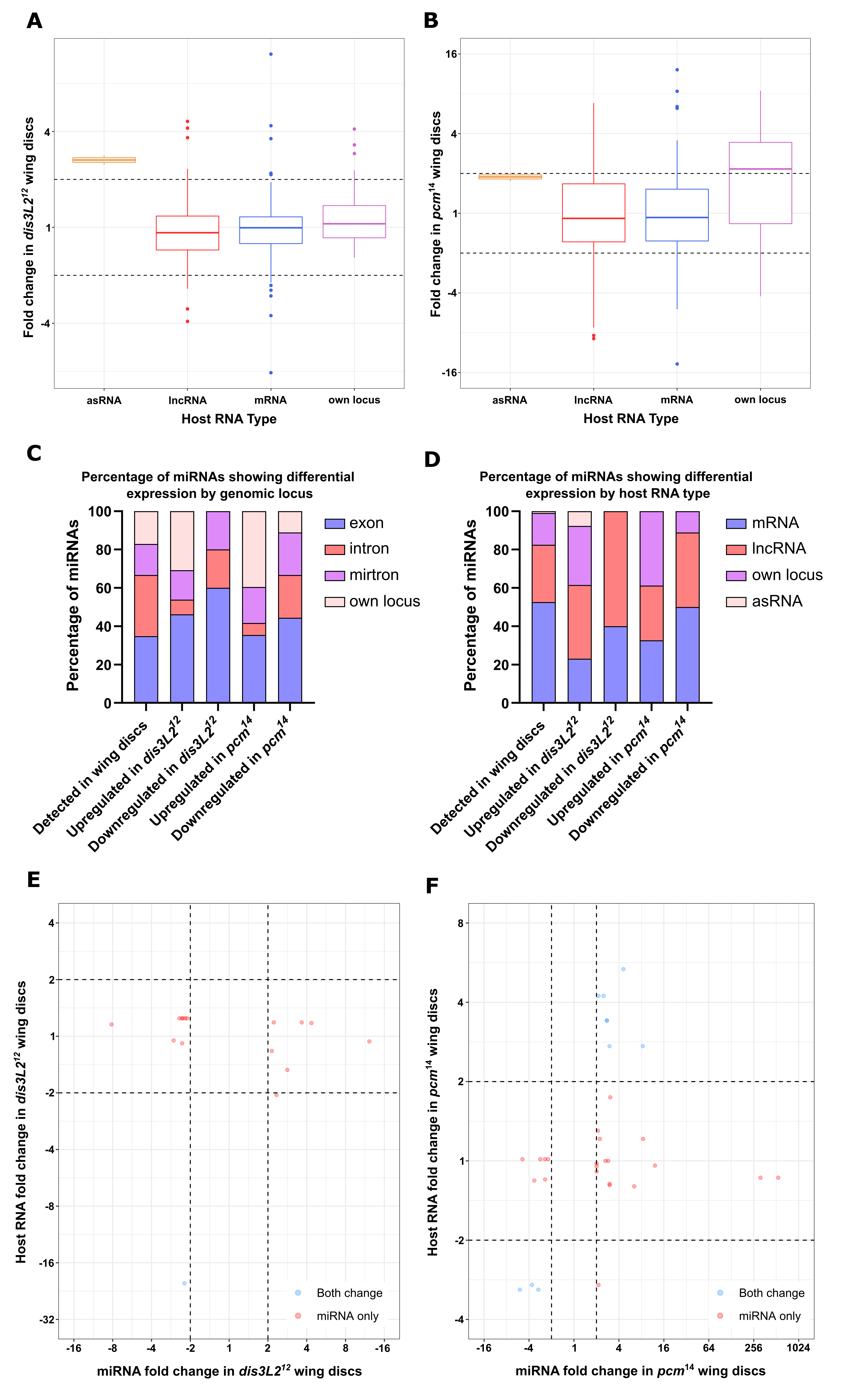

Supplement: Supplementary file 1 [file DataSheet1.zip › SupplementalFig2.tif]

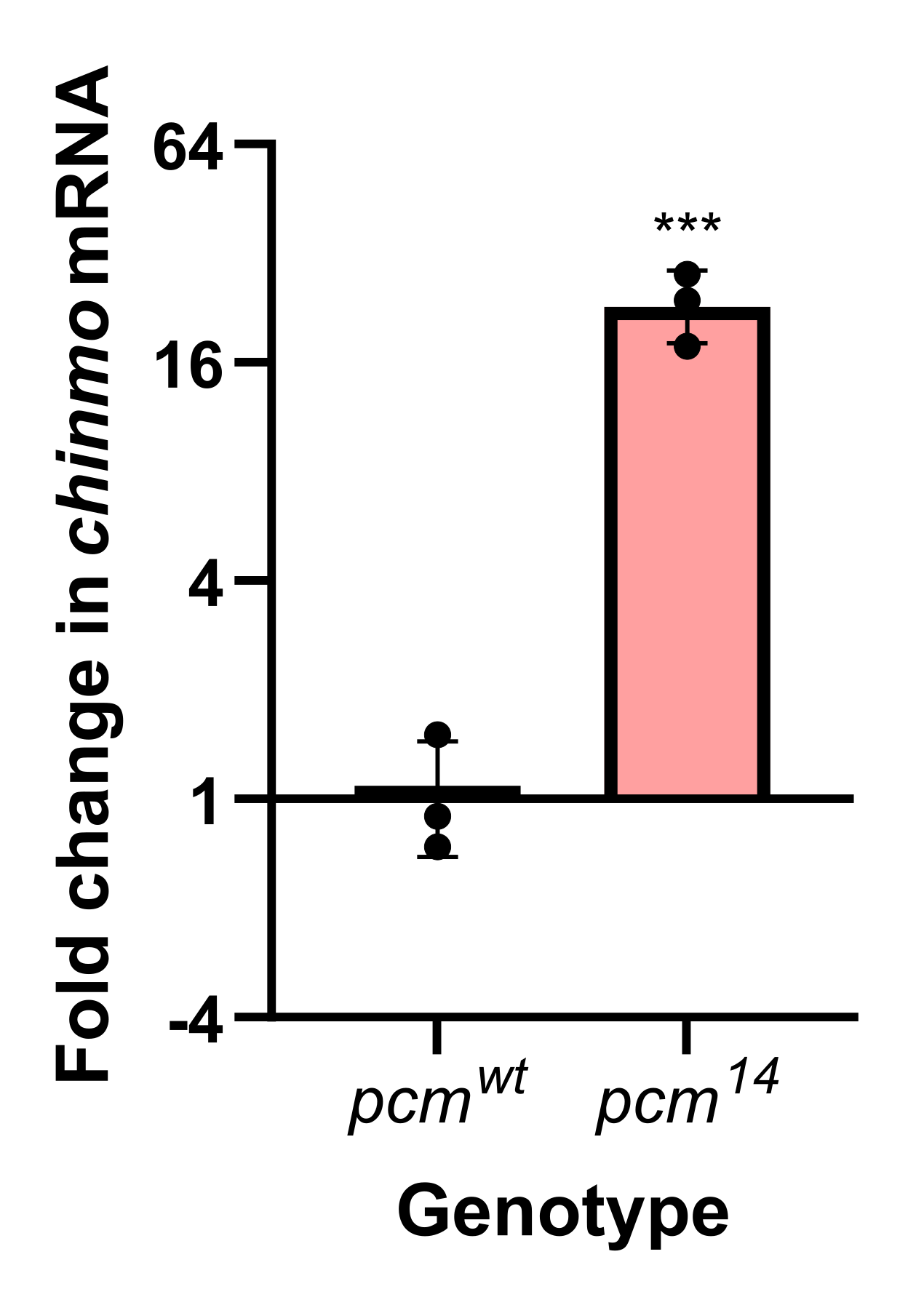

Supplement: Supplementary file 1 [file DataSheet1.zip › SupplementalFig3.tif]
